# Expandable Sendai-Virus-Reprogrammed Human iPSC-Neuronal Precursors: *In Vivo* Post-Grafting Safety Characterization in Rats and Adult Pig

**DOI:** 10.1177/09636897221107009

**Published:** 2023-04-23

**Authors:** Yoshiomi Kobayashi, Michiko Shigyo, Oleksandr Platoshyn, Silvia Marsala, Tomohisa Kato, Naoki Takamura, Kenji Yoshida, Akiyoshi Kishino, Mariana Bravo-Hernandez, Stefan Juhas, Jana Juhasova, Hana Studenovska, Vladimir Proks, Shawn P. Driscoll, Thomas D. Glenn, Samuel L. Pfaff, Joseph D. Ciacci, Martin Marsala

**Affiliations:** 1 Department of Anesthesiology, School of Medicine, University of California, San Diego, San Diego, CA, USA; 2 Department of Orthopedic Surgery, Keio University School of Medicine, Tokyo, Japan; 3 Regenerative & Cellular Medicine Kobe Center, Sumitomo Dainippon Pharma Co., Ltd., Kobe, Japan; 4 Division of Stem Cell Medicine, Department of Advanced Medicine, Medical Research Institute, Kanazawa Medical University, Uchinada, Japan; 5 Institute of Animal Physiology and Genetics, AS CR v.v.i., Liběchov, Czech Republic; 6 Department of Biomaterials and Bioanalogous System, Institute of Macromolecular Chemistry, Czech Academy of Sciences, Prague, Czech Republic; 7 Gene Expression Laboratory, Howard Hughes Medical Institute and Salk Institute for Biological Studies, La Jolla, CA, USA; 8 Department of Neurosurgery, School of Medicine, University of California, San Diego, San Diego, CA, USA

**Keywords:** human induced pluripotent stem cells (hiPSCs), neural precursor cells (NPCs), cryopreservation, spinal cord, human injection device, immunosuppressed adult pig

## Abstract

One of the challenges in clinical translation of cell-replacement therapies is the definition of optimal cell generation and storage/recovery protocols which would permit a rapid preparation of cell-treatment products for patient administration. Besides, the availability of injection devices that are simple to use is critical for potential future dissemination of any spinally targeted cell-replacement therapy into general medical practice. Here, we compared the engraftment properties of established human-induced pluripotent stem cells (hiPSCs)-derived neural precursor cell (NPCs) line once cells were harvested fresh from the cell culture or previously frozen and then grafted into striata or spinal cord of the immunodeficient rat. A newly developed human spinal injection device equipped with a spinal cord pulsation-cancelation magnetic needle was also tested for its safety in an adult immunosuppressed pig. Previously frozen NPCs showed similar post-grafting survival and differentiation profile as was seen for freshly harvested cells. Testing of human injection device showed acceptable safety with no detectable surgical procedure or spinal NPCs injection-related side effects.

## Introduction

Previous preclinical and clinical studies using fetal tissue-derived or embryonic stem cell-derived neural precursor cells (NPCs) have established the rationale for the use of cell-replacement therapies for the treatment of a variety of spinal neurodegenerative disorders^1–3^. In preclinical studies, it was demonstrated, for example, that the spinal grafting of human or rat fetal spinal cord-derived NPCs and human ES-derived NPCs leads to amelioration of spasticity and improvement in motor function in rats with spinal ischemic injury^4–6^. A similar functional benefit was reported using spinal compression or contusion models of spinal traumatic injury in rodents^7–21^. Spinal grafting of human fetal tissue-derived NPCs or glia-restricted precursors has been found to ameliorate neuronal degeneration in rat and mouse models of amyotrophic lateral sclerosis (ALS)^22,23^. In general, consistent long-term engraftment and differentiation to all neural cell types (neurons, astrocytes, and oligodendrocytes) were seen after grafting multilineage NPCs^7,9,11–13,22–25^.

More recently the technology of induced pluripotent stem cells, which permits re-programming of adult somatic cells to the pluripotent stage^26,27^, has opened new opportunities for effective generation of lineage-committed cell lines^28–32^. By using this approach, extensive data were generated which demonstrate similar properties of iPSCs-derived NPCs (compared to fetal tissue or ES-derived NPCs) once grafted *in vivo* in several models of neurodegenerative disorders. Previous studies showed that grafted iPSC-derived NPCs or oligodendrocyte precursor-enriched cells survived and promoted functional recovery after spinal traumatic injury without tumorigenesis in rodents and non-human primates^31,33,34^. Similarly, spinal grafting of iPSC-derived glial-rich NPCs improved survival of ALS SOD1-mice. Grafted cells differentiated preferentially into astrocytes which led to spinal parenchymal upregulation of neurotrophic factors such as VEGF, NT3, GDNF, and increased activity of cell- pro-survival signals such as AKT signaling, which is downstream from the VEGF signal^35^. More recently, it was demonstrated that the iPSC-derived dopaminergic progenitors survived, differentiated toward mature midbrain dopaminergic neurons, and improved neurological deficit after bilateral grafting into putamen in a non-human primate model of Parkinson’s disease^36^.

Some of the preclinical studies which employed fetal, ES-derived NPCs, or human iPSCs-NPCs led to the initiation of human clinical trials in patients with spinal traumatic injury^37,38^, ALS^39,40^ or Parkinson’s disease^41,42^. In general, so far completed or ongoing clinical trials demonstrate acceptable safety profile. While no desired improvement in ambulatory function was achieved so far in spinal trauma patients, some patients demonstrated one to two levels of neurological improvement as assessed by ISNCSCI motor and sensory scores^37^. These early encouraging data warrant further development and testing of cell-replacement technology in human patients with neurodegenerative disorders.

One of the critical determinants for a successful translation of cell-replacement therapies for the treatment of spinal neurodegenerative disorders (as well as brain-associated pathologies) is (1) the establishment of cell-line production protocols which permit access to a readily available, freeze-thaw transplantable cells that can be used for direct patient grafting without additional sub-culturing, and (2) the availability of spinal cell injection device that is simple to assemble and use by trained spinal neurosurgeons.

Accordingly, the goal of the present study was twofold. First, the engraftment properties and safety of established and previously characterized human iPSCs-derived NPCs were tested in the immunodeficient rat. The properties of freshly harvested versus previously frozen/washed NPCs grafted into striata and spinal cord were compared at 2 months post-grafting by immunofluorescence (IF) and differential mRNA sequencing which separate rat-specific versus human-specific mRNA transcripts. Second, a newly developed spinal cell injection device equipped with a spinal pulsation-cancelation magnetic injection needle was tested for acute surgical procedure-related safety in the adult immunosuppressed pig.

## Methods

### hiPSCs-NPCs Culture, NPCs Freezing and Preparation for *in Vivo* Grafting

The previously established hiPSCs-derived NPC cell line was employed in all *in vitro* and *in vivo* NPCs grafting experiments. A detailed description of hiPSC-NPCs generation, expansion, and phenotypic/functional characterization is provided in the accompanying paper (Shigyo et al., accompanying paper).

The generated NPCs were cultured/expanded in poly-L-ornithine/laminin-coated plastic wells using DMEM/F12-based NPC media containing 0.5% N2 supplement (Life Technologies, Carlsbad, CA, USA), 1% B27 supplement (Life Technologies), 1% glutamate (Life Technologies), and 1% penicillin-streptomycin (Life Technologies) supplemented with 10 ng/ml recombinant human basic fibroblast growth factor (bFGF) (Thermo Fisher Scientific, Carlsbad, CA, USA) as a solo mitogen.

#### NPCs freezing

To freeze proliferating NPCs, cells were dissociated with 0.05% trypsin, pelleted (2 x 10^6^ to 6 x 10^6^ cells/pellet), and re-suspended in 1 ml of NPC-proliferating media containing 10% DMSO. DMSO-containing cell suspension was then transferred to 1.5cc cryogenic tubes (Thermo Fisher Scientific, Waltham, MA, USA) and kept in an isopropanol-based freezing container (Thermo Fisher Scientific, Waltham, MA, USA) at -80C overnight. The next day the cryovials were transferred into liquid nitrogen vapor (-150°C) for long-term storage.

#### Preparation of NPCs for *in vivo* grafting

Two different NPC preparation protocols were used. First, *in vitro* proliferating NPCs (=Fresh NPCs) were washed with DPBS and dissociated with 0.05% Trypsin EDTA (Thermo Fisher Scientific) at 37°C, 5% CO_2_ incubator for 2–3 min. After adding the Trypsin-inhibitor (Thermo Fisher Scientific, Waltham, MA, USA), cell suspensions were collected in a 15-ml tube and centrifuged for 4 min at 400*g*. After discarding the supernatant, the pellet was re-suspended-washed in hibernation buffer (Thermo Fisher Scientific, Waltham, MA, USA) and centrifuged again (400*g*) for 4 min. After washing, the final cell pellet was re-suspended in hibernation media, filtered through 40 μm plastic mesh (Thermo Fisher Scientific, Waltham, MA, USA), and the desired cell density solution was prepared. Second, previously frozen NPCs (stored in LN vapor; =Frozen NPCs) were thawed quickly in the water bath (37°C) and transferred to 15cc of hibernation media for wash (Thermo Fisher Scientific, Waltham, MA, USA). The cell pellet was then re-suspended with a hibernation medium and filtered through 40 μm plastic mesh (Thermo Fisher Scientific, Waltham, MA, USA) and targeted cell density solution prepared.

### *In Vitro* NPCs Differentiation and Indirect Immunofluorescence

Proliferating or previously frozen-washed NPCs, were plated onto glass chamber slides (Thermo Fisher Scientific, Waltham, MA, USA) and induced to terminally differentiate by the withdrawal of bFGF and adding 10 ng/ml BDNF (Peprotech, Rocky Hill, NJ, USA) and 10 ng/ml GDNF (Peprotech, Rocky Hill, NJ, USA) into culture media for 2 weeks. After differentiation, cells were fixed with 4% paraformaldehyde, stained with neuronal and glial markers, and images captured and analyzed with a Fluoview FV1000 confocal microscope (Olympus, Center Valley, PA, USA). All primary and secondary antibodies are listed in Table 1.

**Table 1.** Antibodies Used for Immunofluorescence Staining.

Primary antibodies used for immunofluorescence staining		
Catalog #	Name	Company
MAB6326	Nestin	Millipore Sigma
S4403	MAP2	Sigma
AB5733	Vimentin	EMD Millipore
SC-8066	DCX	Santa Cruz biotechnology
Ab16667	Ki67	abcam
MMS-435P	TUJ	Covance
Ab36999	Human NuMA	abcam
TA302094	Human GFAP	Origene
134014	NeuN	Abcam
MAB377	NeuN	Millipore Sigma
ABN78	NeuN	Millipore Sigma
Ab9610	Olig2	Millipore Sigma

### *In Vivo* Cell Grafting

All animal studies were approved by the University of California, San Diego Institutional Animal care and Use Committee (Protocol No. S01193). First, the adult athymic rats (Crl: NIH-Foxn1^rnu^; Charles River, Wilmington, MA, USA) were used for intrastriatal and spinal hiPSC-NPC grafting in the rodent component of the *in vivo* grafting study (n = 7). To graft fresh and previously frozen iPSC-NPCs spinally, the previously described technique was used^4,22^. Animals received 10 to 15 spinal NPC injections (0.5 µl each) distributed bilaterally between L2 and L6 spinal segments (20,000–30,000 viable cells per injection; depth of injections from dorsal spinal cord surface: 1 mm) using a homemade glass capillary (inner diameter: 80 µm, outer diameter: 100 µm). Each animal received both fresh and frozen NPCs injected either into the left or right side of the spinal cord, respectively. Each animal received an identical number of fresh and frozen NPCs injections. In addition to spinal cord injections, the same animals received a bilateral NPC injection (Fresh or Frozen NPCs) into the striatum. To perform brain injections, anesthetized animals were placed into a stereotaxic apparatus to keep the head in a fixed position. After the scalp was shaved, a sagittal midline skin incision was performed to expose the skull. To permit an intra-parenchymal brain injection a small borrow hole was drilled into the skull using a dental drill. The NPCs (30,000 cells/µl/injection) were injected into the striatum (stereotaxic X-Y-Z coordinates: bregma 0.5 mm, lateral to ± 3.0 mm and 3.8 mm, 5.0 mm, and 6.2 mm depth: 3 injections delivered at each depth bilaterally) using a 34G needle interconnected with a digital microinjector (Tritech Research, San Diego, CA, USA).

After cell grafting rats survived for 2 months (n = 4) or 6 months (n = 3). At sacrifice, animals were terminally anesthetized and transcardially perfusion-fixed with 4% paraformaldehyde (n = 4; 2 months survival; n = 1; 6 months survival; see Perfusion Fixation) or brain and spinal cord tissue harvested fresh and snap-frozen for mRNA sequencing (n = 2; 6 months survival; see RNA sequencing).

Second, adult minipig model was employed for spinal cell grafting (n = 3). Adult female Gottingen-Minnesota minipigs were anesthetized and the L2-L3 spinal segment was exposed after partial dorsal laminectomy of the L2-3 vertebra as previously described^43^. Dura was then cut open. Animals then received a total of 10 injections of Frozen-hiPSC-NSCs (10 µl/injection; 20,000–30,000 cells/µl; injection flow rate = 2 µl/min; depth of injections from dorsal spinal cord surface: 4 mm). From the day of the cell grafting, animals were continuously immunosuppressed by tacrolimus (0.6 mg/kg/day). After cell grafting animals survived for 2 days.

### Perfusion Fixation, Indirect Immunofluorescence Staining of Brain and Spinal Cord Sections and Quantitative Analysis of Grafted Cell Neuronal and Glial Differentiation

At the end of survival, rats were anesthetized with 2 mg pentobarbital and 0.25 mg phenytoin (0.5 mL of Beuthanasia-D, Intervet/Schering-Plow Animal Health Corp., Union, NJ, USA) and transcardially perfused with 200 ml of heparinized saline followed by 250 ml of 4% paraformaldehyde (PFA) in PBS. Brain and spinal cord sections were then prepared and stained with a combination of human-specific and non-specific antibodies (Table 1) as previously described^44^.

For quantitative and qualitative analysis, brain and spinal cord sections taken from immunodeficient rats at 2 months after hiPSC-NPC grafting were used. In qualitative and quantitative analysis of combined antibodies-stained sections, six sections (three brain and three spinal cord sections) taken from each animal with identified grafts were used. Sections were stained with hNUMA antibody in combination with neuronal and glial markers including DCX, NeuN, hGFAP, vimentin, Olig2, and Ki67. The total number of double-stained grafted cells was then counted and expressed as % of the total hNUMA stained cell population. Quantitative analysis of grafted hNUMA+ cells-occupied regions in striatum and spinal cord was performed on minimum of 10 sections/animal (five brain and five spinal cord sections) and expressed as hNUMA+ area in mm^2^.

On the day of sacrifice, pigs were deeply anesthetized with pentobarbital (100 mg/kg) and transcardially perfused with ice-cold, heparinized (4 U/ml; 5L) PBS (137 mM sodium chloride, 2.7 mM potassium chloride, 8 mM sodium phosphate dibasic, and 1.47 mM potassium phosphate monobasic), followed by 4% paraformaldehyde (5 L; pH 7.4). The spinal cords were then removed and postfixed for 24 h in 4% paraformaldehyde at 4°C. After postfixation, spinal cord tissue was cryoprotected in 10%, 20%, and 30% sucrose with 0.02% sodium azide and 10- to 40-µm-thick sections cut on a cryostat. Staining protocol is identical as described for NPCs-grafted rat.

### mRNA Sequencing and Data Analysis

Total RNA was isolated from flash frozen grafted spinal cord and striatum samples (6 months post-grafting) using the Qiagen RNeasy Plus Mini kit. Approximately 1 to 2 μg of RNA was isolated from each sample, with RIN scores of ~9.0, as determined by TapeStation analysis (Agilent, Santa Clara, CA, USA). mRNA sequencing libraries were prepared using the Illumina Stranded mRNA Library kit, and sequenced at the UC San Diego IGM Genomics Center, using the Illumina NovaSeq 6000 platform. Paired-end 100bp reads were received in FASTQ format and were prepared for downstream analysis by trimming known barcodes and then selecting post-trimmed reads for those with average base quality >15. The assignment of transcripts to species of origin (human or rat) was performed using our previously published methods^24^. Reads were aligned with HISAT2 to a combined rat (Rn6) and human (Hg38) genome index. We used -k 20 and—score- min L,0,-0.4 with otherwise default settings. Aligned reads were sorted by read name and then evaluated for species of origin with an in-house Perl script. The vast majority of reads only aligned to a single genome, but for those that aligned to both genomes we used the HISAT2 alignment score to guide species assignment. If a read mapped equally well to both genomes then it was flagged as ambiguous and excluded from downstream analysis. Otherwise, the read was assigned to the species for which it had the best alignment score. Species assigned reads were quantified using Kallisto. Human reads were quantified against the full Gencode annotation (v28) and rat assigned reads were quantified against the Refgene annotation for the Rn6 genome (obtained from the UCSC Genome Browser). Kallisto “quant” was run in single-end mode with average fragment length of 200 and SD of 60. All downstream analysis was performed in R. Human and rat quantified expressions were combined into a single table and treated as a single-species for normalization. After calculating TPM scaled expression, we split the human and rat gene-sample read count matrices apart for further analysis.

### Neurological Assessment

After cell grafting rats were evaluated periodically (in 2 weeks intervals) for any signs of motor dysfunction (potentially indicative of grafted-cell-expansion-induced spinal cord compression) using BBB scoring system^45^. To assess the degree of motor dysfunction in cell-grafted pig, previously developed 14-grades scoring system was used^46^. In addition, exacerbated pain response to non-noxious mechanical stimuli such as light skin brush was tested at 48 h after cell grafting (ie at the time point of sacrifice).

### Statistical Analysis

All data are reported as the mean ± SEM. An unpaired two-tailed Student’s t-test was used for single comparisons between the grafted Fresh-hiPSC-NPCs and the grafted Frozen-hiPSC-NPCs for all histological examinations. In each case, *P < 0.05 and **P < 0.01 were considered to be statistically significant. The GraphPad Prism software (version 6.0c; GraphPad Software Inc., San Diego, CA, USA) was used for all analyses.

## Results

### Experimental Design Overview

**Figure 1.**
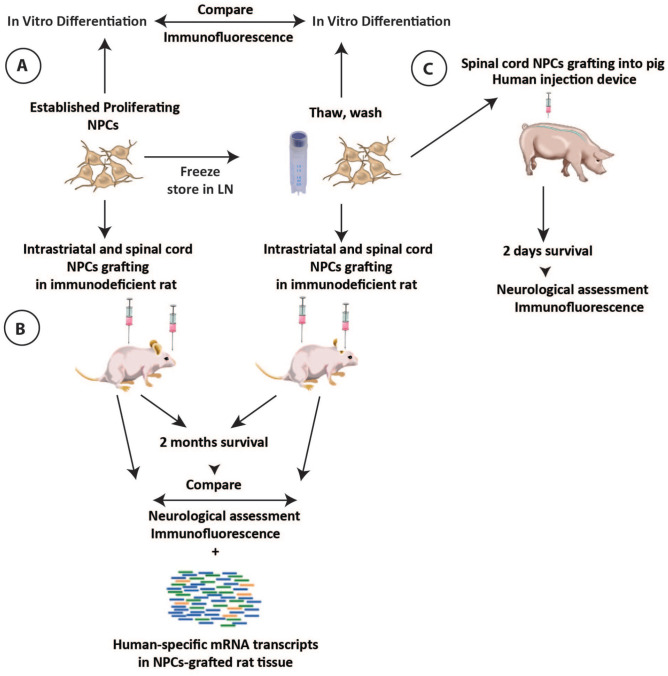
Schematic diagram of the experimental design. (A) Established *in vitro* proliferating hiPSCs-derived NPCs or previously frozen hiPSCs-NPCs is thaw and washed and induced *in vitro* to differentiate for 14 days. After differentiation, the phenotype of induced cells is studied by qualitative and quantitative IF. (B) *In vitro* proliferating NPCs or previously frozen, thaw and washed NPCs and injected into striata and spinal cord of the immunodeficient rat as a single-cell NPCs suspension. Every animal receives both freshly harvested or previously frozen NPCs. After cell grafting animals survive for 2–6 months and the presence of grafted cells analyzed using IF and differential mRNA sequencing which recognizes human-specific transcripts. (C) Previously frozen, thaw, and washed NPCs are injected into the lumbar spinal cord of immunosuppressed adult pig. To inject NPCs into spinal cord parenchyma a novel human spinal injection device is used. After cell grafting animals are evaluated for acute injection-related toxicity for 2 days. The presence of spinally grafted NPCs is evaluated by IF. hiPSCs: human induced pluripotent stem cells; NPC: neural precursors cell; IF: immunofluorescence; LN: liquid nitrogen.

Previously established human iPSCs-derived neuronal precursors at passages 9-11 were used (Shigyo et al., accompanying paper). First, continuously *in vitro* cultured-proliferating or previously frozen NPCs were induced to differentiate for 2 weeks *in vitro* and the differentiation profile compared using quantitative and qualitative IF, (Fig. 1A). Second, the same NPCs population (ie freshly harvested proliferating NPCs or frozen and washed NPCs) were grafted into striata and spinal cord of the immunodeficient rat. After grafting animals survived for 2 to 6 months and the engraftment properties analyzed by IF and mRNA sequencing of human-specific transcripts (Fig. 1B). Third, previously frozen NPCs (stored in liquid nitrogen) were thawed, washed, and injected into the lumbar spinal cord of immunosuppressed adult pig using a human injection device. After injections, animals survived for 2 days while being assessed for the recovery of motor and sensory function. The presence of grafted cells was validated with IF staining (Fig. 1C).

### *In Vitro* Proliferating and Induced and Previously Frozen and Induced NPCs Differentiate Into Neurons and Glia *in Vitro*

**Figure 2.**
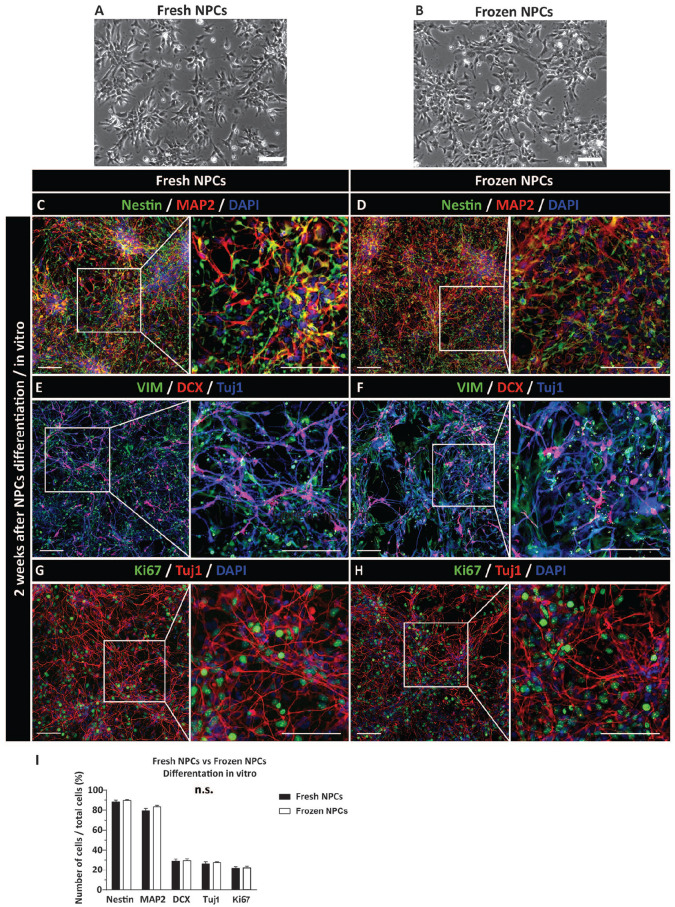
Similar differentiation profile of induced-proliferating NPCs or previously frozen and induced NPCs *in vitro*. (A, B) Bright field morphology of proliferating and previously frozen-washed and plated NPCs. The image from previously frozen cells was taken at 48 h after plating in NPC media. (C, D) Co-staining with Nestin and MAP2 antibodies shows a mixed population of Nestin+, MAP2+, or Nestin/MAP2 expressing cells. (E, F) Presence of early postmitotic DCX+ neurons and early glial VIM + precursors. (G, H) Staining with the Tuj1 antibody show well-developed neurons with axodendritic arborization. In the same field, a high density of mitotically active cells (Ki67+) is also seen. (I) Quantitative analysis of Nestin, MAP2, DCX, Tuj1, and Ki67-positive cells showed no significant difference between freshly induced and frozen-washed and induced NPCs. Scale bars: 200 µm (A–H). NPC: neural precursors cell; VIM: Vimentin; DAPI: 4′,6-diamidino-2-phenylindole.

Using a brightfield microscopy, a similar morphological appearance of continuously cultured NPCs versus freshly plated NPCs from frozen stock (2 days post-plating) was observed prior to the differentiation (Fig. 2A, B).

Proliferating and induced or previously frozen and induced NPCs were analyzed with neuron-specific and glia-specific markers at 2 weeks after *in vitro* induction using differentiation media (see M&M for details). Staining with nestin (proliferating NPCs marker) and MAP2 (early neuronal marker) antibodies showed a high density of nestin/MAP2-stained cells, suggesting an early stage of neuronal differentiation (Fig. 2C, D). Staining with mature astrocyte marker GFAP showed no positive cells (**not shown**), while a relatively high density of vimentin+ cells (early glial marker) was seen (Fig. 2E, F). A consistent homogenous DCX expression (early postmitotic neuronal marker) and Tuj1 (neuronal marker) throughout the whole population of induced NPCs was seen (Fig. 2E–H). Probing for the presence of mitotically active cells showed a high density of Ki67+ cells (Fig. 2G, H). Qualitative and quantitative comparison between NPCs induced from active-proliferating culture versus previously frozen and directly induced to differentiate after thawing showed no detectable differences (Fig. 2I).

### Previously Frozen NPCs Grafted Into CNS of Adult Immunodeficient Rats Show Similar Survival and Differentiation as Freshly Harvested and Grafted NPCs

We next compared the survival and differentiation of freshly harvested versus previously frozen and washed NPCs after grafting into striata and spinal cord of immunodeficient adult rat. First, *in vitro* proliferating NPCs were dissociated with trypsin and single-cell suspension prepared. Second, previously frozen NPCs stored in liquid nitrogen vapor were thawed, washed in hibernation buffer, and single-cell suspension prepared. A similar morphological appearance of single-cell NPCs suspension was observed when comparing freshly harvested or previously frozen-washed NPCs (Supplemental Fig. 1A, B). The viability (trypan blue extrusion test) ranged between 93% and 94% for freshly harvested NPCs and was between 88% and 97% for previously frozen and washed NPCs (Supplemental Fig. 1C). Each animal received three bilateral intrastriatal injections, and 10 lumbar spinal cord injections of both freshly harvested and previously frozen-washed NPCs targeted at ipsi- or contralateral site, respectively. After cell injection, animals survived for 2 months and the presence of grafted cells studied using a combination of human-specific or non-specific neuronal or glial antibodies.

Neurological assessment (BBB score) showed no detectable motor dysfunction for the duration of 2-6 months of survival. Quantitative comparative analysis of hNUMA+ cells in striata and spinal cord (defined by the surface area occupied by hNUMA+ calls), showed similar grafts size in both grafted cell population with no significant difference between freshly harvested or previously frozen-washed NPCs (Supplemental Fig. 2A–F).

**Figure 3.**
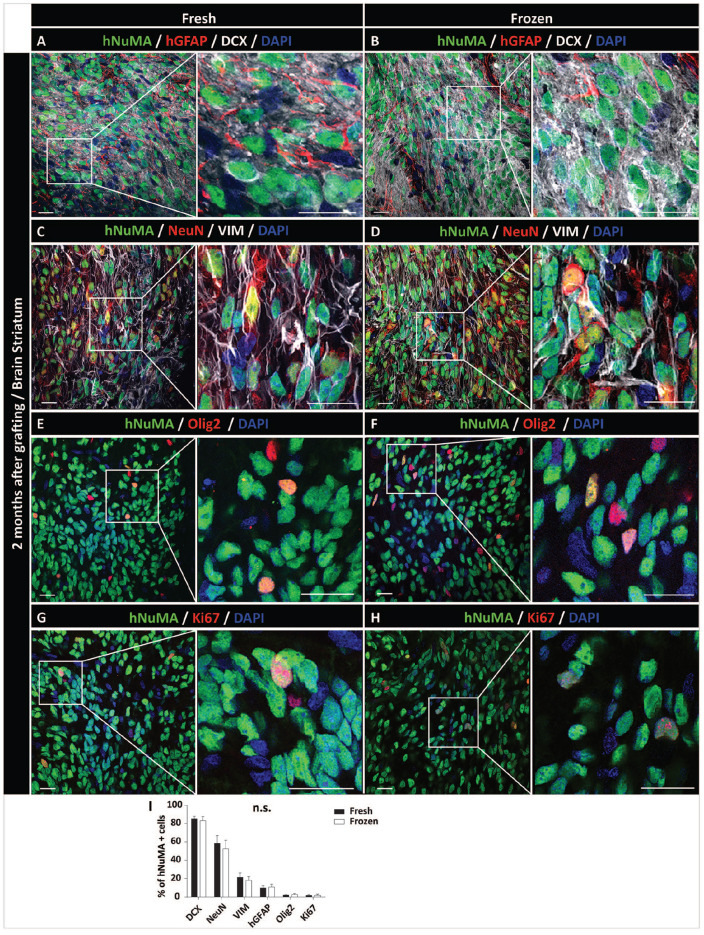
Comparable survival and differentiation of freshly harvested or previously frozen-washed NPCs grafted into striata of the immunodeficient rat. (A, B) Comparable engraftment of hNUMA + cells with DCX and hGFAP immunoreactivity in the core of the graft. (C, D) Expression of NeuN in hNUMA+ neurons. Homogenous vimentin IF in the same areas can also be seen. (E, F) Sparse presence of grafted cells-derived oligodendrocytes (Olig2). (G, H) Co-expression of Ki67 mitotic marker in grafted hNUMA cells. (I) Quantitative analysis of neuronal, glial, and mitotic marker expression in grafted cells. No significant differences in differentiation profile between NPCs grafted as freshly harvested or previously frozen cells can be seen. Scale bars: 20 µm (A-H), 10 µm (A-H inserts). NPC: neural precursors cell; VIM: Vimentin; DAPI: 4′,6-diamidino-2-phenylindole; hGFAP-human-specific glial fibrillary acidic protein.

**Figure 4.**
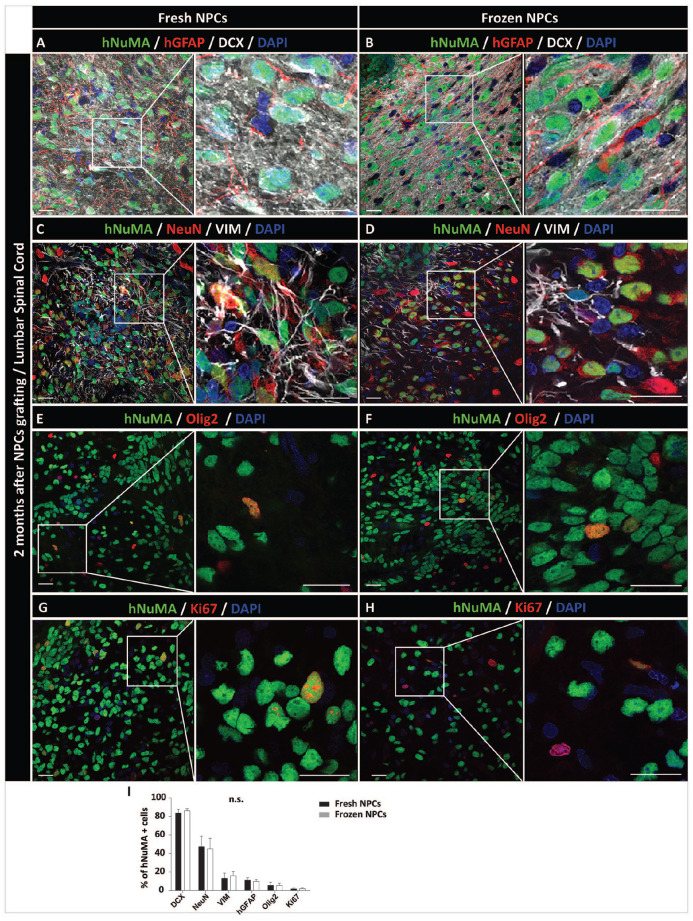
Survival and differentiation of freshly harvested or previously frozen-washed NPCs grafted into the lumbar spinal cord of the immunodeficient rat. (A, B) Presence of hNUMA+ cells with dense DCX immunoreactivity in early postmitotic neurons. hGFAP immunoreactivity throughout the grafts can also be seen. (C, D) NeuN expression in mature neurons in the core of hNUMA+ grafts. Vimentin positivity was typically seen at the periphery of the grafts. (E, F) Presence of grafted cells-derived oligodendrocytes (Olig2) throughout the grafts. (G, H) The occasional presence of mitotically active cells (Ki67) in hNUMA + grafts. (I) Quantitative analysis of neuronal, glial, and mitotic marker expression in grafted cells. No significant differences between NPCs grafted as freshly -harvested or previously frozen cells were identified. Scale bars: 20 µm (A-H), 10 µm (A-H inserts). NPC: neural precursors cell; VIM: Vimentin; DAPI: 4′,6-diamidino-2-phenylindole; hGFAP-human-specific glial fibrillary acidic protein.

Qualitative analysis of neuronal and glial markers in NPCs-grafted striata and spinal cord showed a very similar pattern in both NPCs-grafted population (ie freshly harvested and freeze-wash NPCs). Intense immunoreactivity was seen for early post-mitotic neuronal marker DCX as well as more mature neuronal marker NeuN (Figs. 3A–D and 4A–D). Staining with human-specific mature astrocyte marker GFAP and vimentin (early glial marker) showed a homogenous distribution of hGFAP+ astrocytes throughout the individual grafts (Figs. 3A, B and 4A, B) and with a higher density of vimentin+ cells (Figs. 3C, D and 4C, D). Expression of oligodendrocyte marker Olig2 was seen in a relatively small number of grafted hNUMA+ cells (Figs. 3E, F and 4E, F). Probing for the presence of mitotically active grafted cells showed the only occasional presence of double-stained Ki67/hNUMA+ cells (Figs. 3G, H and 4G, H). Quantitative analysis of neuronal and glial marker(s) expression showed no detectable differences in grafts derived from freshly harvested vs. previously frozen and directly grafted NPCs (Figs. 3I and 4I).

### mRNA Sequencing Analysis Demonstrates That Freshly Harvested and Previously Frozen NPCs Give Rise to Neurons and Glia, and Have Similar Differentiation Profiles 6 Months After Transplantation

**Figure 5.**
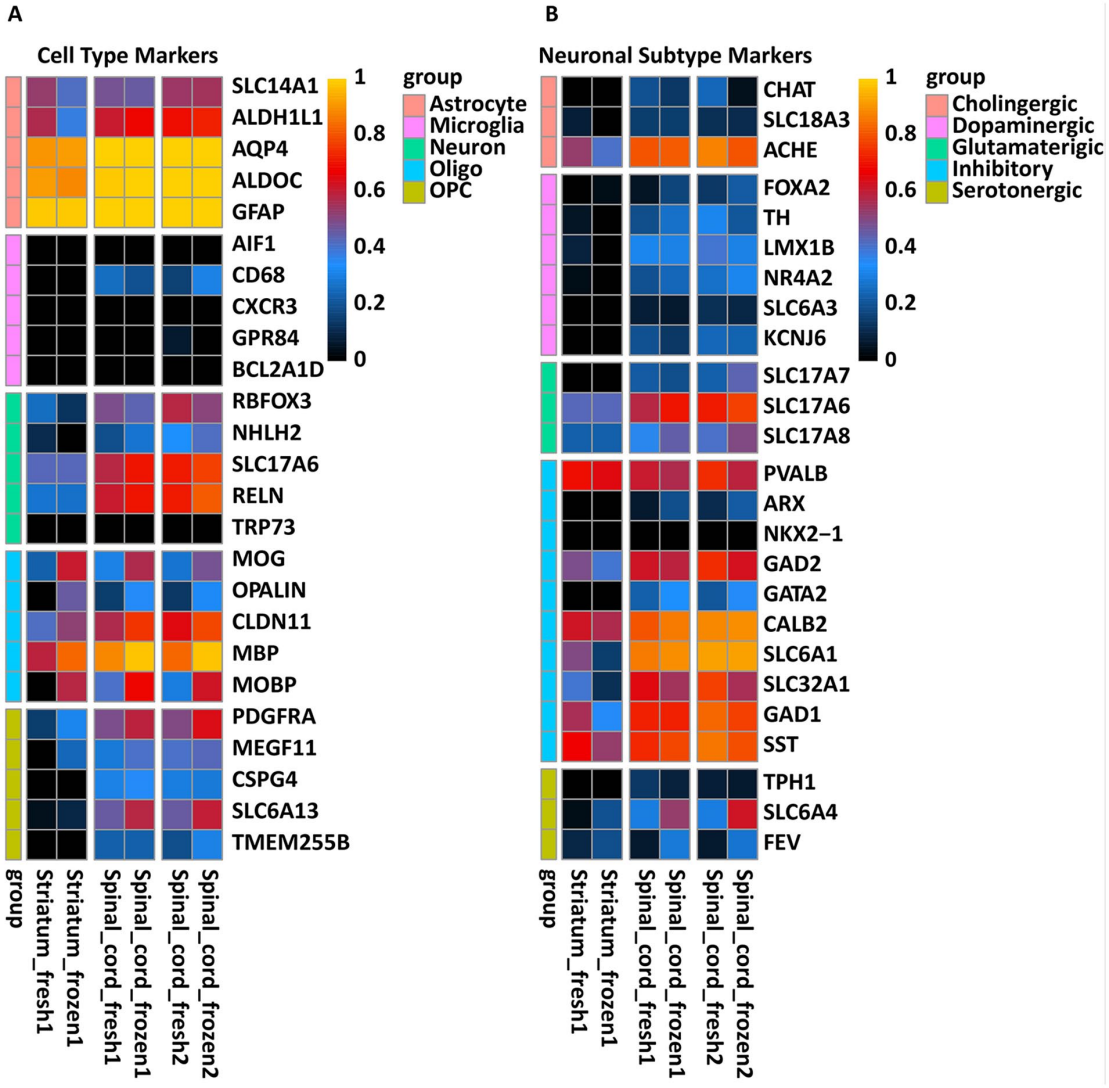
Expression of human-specific neuronal and glial lineage transcripts in rat striata and spinal cord 6 months after NPCs grafting. (A) Heat map showing the expression of an array of cellular markers for astrocytes, microglia, neurons, oligodendrocytes (oligo) and OPCs. Widespread expression of markers from neural progenitor cell-derived lineages is observed, with minimal or no expression of microglia markers. (B) Heat map showing the expression of an array of neuronal subtype markers, including cholinergic, dopaminergic, glutamatergic, inhibitory, and serotonergic markers. All heat maps are normalized according to the average gene expression across all samples, and only human specific transcripts are represented here. NPC: neural precursors cell; OPCs: oligodendrocyte precursor cells.

In order to analyze the differentiation profiles of engrafted NPCs 6 months post-transplantation, we dissected tissue samples from the striatum and spinal cord that contained transplanted cells. mRNA sequencing was performed on this chimeric tissue, and reads were assigned to the correct species of origin (rat vs. human) using the bioinformatics protocols we have developed previously^24,47^. An array of known cellular markers was used to profile the human-derived transcripts. Consistent with the immunofluorescent analysis of spinal cord and striatal grafts, we detected the clear presence of human-specific transcripts corresponding to neuronal, astrocyte, and oligodendrocyte lineage markers (Fig. 5A). We next probed for markers of specific neurotransmitters and neuronal subtype identity, which indicated that grafted NPCs had differentiated into multiple neuronal subtypes, including both inhibitory and excitatory neurons (Fig. 5B). Multiple inhibitory neuron-associated genes were detected (e.g. GAD65/GAD2, GAD67/GAD1, SLC6A1, SLC32A1), as well as strong expression of the glutamatergic marker SLC17A6 (VGLUT2), particularly in the spinal cord. We also detected evidence of cholinergic, dopaminergic, and serotonergic neurons, with expression of these subtypes being more prevalent in the spinal cord grafts (Fig. 5B). Analysis of germ layer markers confirmed the presence of ectoderm-associated genes, including OTX2, Nestin, and TUBB3 (βIII-tubulin), and a very low (or undetectable) level of transcripts that mark mesodermal and endodermal tissue (Supplemental Fig. 3A). An analysis of leukocyte markers (the cells used for reprogramming and generation of iPSCs) demonstrated a low level of expression for CD14 and FUT4 (CD15), with undetectable levels of other markers (Supplemental Fig. 3B). Analysis of the reprogramming factors (SOX2, OCT4, KLF4, and MYC) showed undetectable levels of OCT4, low levels of expression of KLF4 and MYC, and high levels of expression for SOX2. This strong expression of SOX2 is indicative of the persistence of immature neuronal precursors in the grafted tissue (Supplemental Fig. 3C).

### Safety of Human Spinal Cell Injection Device in an Adult Immunosuppressed Pig

**Figure 6.**
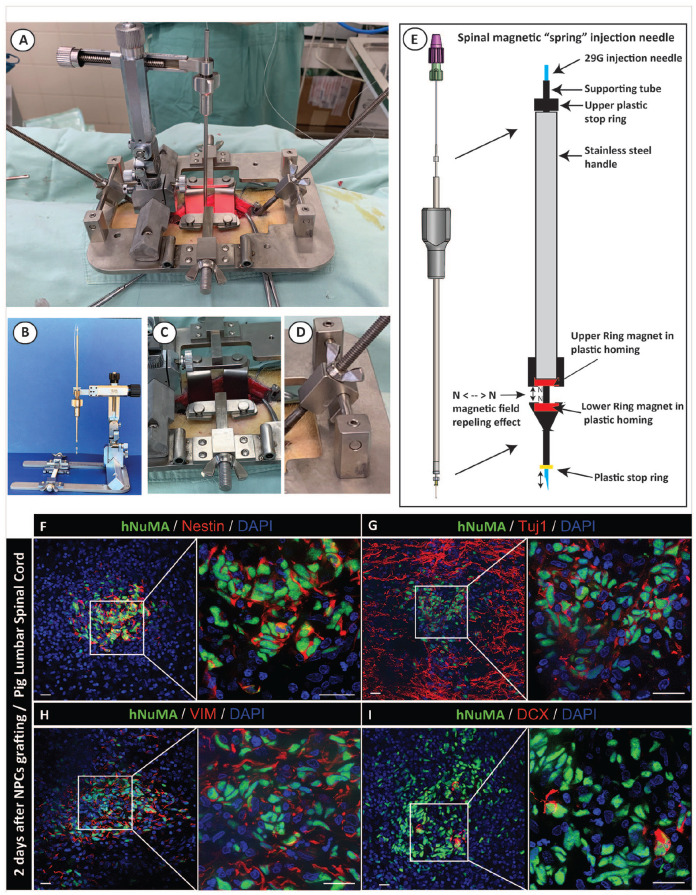
Safety and use of novel human spinal cell injection device in the adult pig. (A, B) The human spinal injection system is composed of the spinal platform, XYZ manipulator, and injection needle and mounted over the laminectomy site in the adult pig. (C, D) The spinal platform is equipped with bilateral muscle retractors (C) and spinal clamps which are attached to the side of the platform (D) and clamped to the spinal processes. (E) Cells are injected into the spinal parenchyma using a spinal cord pulsation-cancelation magnetic needle. The spinal cord pulsation-cancelation effect is achieved by creating a magnetic repelling effect by positioning two ring magnets at the tip of the needle and which are oriented with the identical magnetic N< - > N field toward each-other. (F, G, H, I) Presence of grafted hNUMA+ cells in targeted intermediate zone seen in lumbar spinal cord sections. Grafted hNUMA+ cells showed co-expression of nestin (F), Tuj1 (G), vimentin (H), and DCX (I). Scale bars: 100 µm (F-I). VIM: Vimentin; DAPI: 4′,6-diamidino-2-phenylindole.

The human spinal injection device was designed and constructed from three separate components (Fig. 6A–E). First, a stainless steel self-anchoring spinal platform equipped with bilateral muscle retractors and spinal clamps (Fig. 6C, D) is used for the surgical exposure of the laminectomy site and a firm attachment of the XYZ manipulator to the patient vertebral column. A firm attachment of the XYZ manipulator to the spinal column eliminates any potential needle misplacement during spinal injection which can be caused by the movement of the patient body. Second, the XYZ manipulator attached to the spinal platform using a crossing stainless steel bar (Fig. 6A, B) holds the spinal magnetic injection needle (Fig. 6A, B, E) and permits a single-hand-controlled spinal injection. Third, the spinal injection needle (27-30G) is equipped with the “magnetic spring” needle tip. This system permits a free movement of the needle previously placed into spinal parenchyma and which follows the dorso-ventral pulsation of the spinal cord. This spring effect is achieved by creating a magnetic-repelling effect by positioning two ring magnets at the tip of the needle and which are oriented with the identical magnetic N< - > N field toward each-other (thus causing repelling-spring effect), (Fig. 6E).

A transplantation protocol likely to be used in a clinical setting will employ previously frozen hiPSC-NPCs stored in clinical cell banks. In a recently completed ALS trial^48^ and our ongoing spinal trauma trial^37^, clinical-grade human fetal spinal cord-derived NPCs previously stored in liquid nitrogen (LN) were washed and shipped in hibernation buffer at 4°C to the clinical site. After a viability test was performed (the cut-off was 70% viability), the NPCs were used directly for spinal grafting without sub-culturing.

To test this likely cell-preparation-grafting scenario, the hiPSC-NPCs generated in our current study were stored-frozen in LN for 4 weeks. On the day of grafting, the hiPSC-NPCs were washed 3x in hibernation buffer, viability test performed and cells were kept at 4°C for 2–3 h prior to being used for *in vivo* grafting.

To perform spinal cell injection in adult pig, a dorsal laminectomy of L3-L4 vertebra was performed and the human injection device mounted over the laminectomy site (Fig. 6A, C). Dura was cut-opened and secured to the surrounding paravertebral muscle using 5.0 Proline-monofilament. Animals then received a total of 10 bilateral injections (5 on the left- and five on the right side of the spinal cord) of NPCs delivered in 6 µl of buffer (30,000cells/µl) using 0.5 µl/min infusion rate. The depth of the parenchymal penetration needle tip was 4 mm and targeting the intermediate zone (~lamina VII). Animals were immunosuppressed with tacrolimus (0.025/kg/day) delivered in microsphere formulation sc^44^. After cell injections, animals survived for 2 days while being evaluated neurologically for any signs of motor deficit and allodynia (exacerbated pain response to non-noxious mechanical stimuli such as light skin brush). After 2 days, animals were perfusion-fixed with 4% paraformaldehyde and the presence of grafted cells in spinal cord sections confirmed with IF.

All cell-grafted animals showed normal ambulatory motor function with no overt signs of motor weakness, muscle spasticity, or allodynia. Staining of spinal cord sections with hNUMA and nestin antibodies showed the presence of nestin+ grafted NPCs (Fig. 6F). This was expected due to the early post grafting time point (ie 2 days) analyzed. The location of individual hNUMA+ grafts was consistently found in the targeted central gray matter regions. Due to the early time point after NPCs grafting staining with neuronal markers (TUJ1 and DCX) showed relatively moderate expression while more homogeneous expression of early glial marker vimentin was seen (Fig. 6G–I). Overall, all individual grafts showed very good incorporation into the host tissue with no injection procedure-associated tissue injury such as peri-injection tissue necrosis or edema. Besides, a quite extensive hNUMA+ cell migration from the core of the grafts, extending for up to 500-800 µm from the injection core into the host tissue was already seen (Fig. 6I).

## Discussion

By using previously established and characterized human iPSCs-derived NPCs (Shigyo et al., accompanying paper), we directly compared the survival, differentiation, and safety/tumorigenicity of *in vivo*-grafted NPCs once cells were harvested fresh from proliferating NPCs culture or prepared from previously frozen/washed NPCs stock and grafted into striata and lumbar spinal cord of the immunodeficient rat. By using a novel human spinal cell injection system, we also studied the safety of spinal cell injection procedure in adult pig receiving multiple spinal injections of NPCs. Overall, we demonstrate that independent of the format how the NPCs were prepared before grafting (ie freshly harvested from *in vitro* proliferating NPCs or previously frozen/washed and grafted NPCs), grafted NPCs show robust engraftment and neuronal and glial differentiation. No detectable tumor formation was seen between 2 and 4 months post grafting. No injection procedure-related side effect (such as motor weakness or allodynia) was seen in spinally grafted pigs with NPCs grafts identified in targeted gray matter regions (Laminae III-VII).

### Defining the Optimal NPCs Derivation and Expansion Protocol for Generation of Clinical Grade NPCs

One important requirement in developing a clinical CNS cell-replacement therapy protocol(s) is the ability to consistently deliver a targeted cell dose to the injection site. As such, the format of how the cells are cultured, stored, and prepared for *in vivo* transplantation (ie single-cell suspension or neurospheres) is important as it defines the level of precision how the desired cell dose can be delivered. We have opted for the use of *in vitro* cell monolayer NPCs culture to expand established human iPSCs-derived NPCs. As described in the accompanying paper (Shigyo et al., accompanying paper), the use of cell culture monolayer system has several specific advantages including (1) high consistency in expanding morphologically defined, homogenous population of NPCs which maintain the expression of neural precursors markers and *in vitro* differentiation-defined multipotency, (2) ability to effectively prepare a single cell suspension with a minimal degree of cell clumping, and (3) reliable cell freezing and cell recovery after freeze/thaw procedure. As such, the use of this protocol, defined by reliable and predictable cell expansion and post-freezing cell recovery in a single cell suspension format makes this approach a preferable technology for the generation of clinical-grade NPCs.

Several previously completed human trials have employed similar cell-derivation and cell-freezing-recovery protocols. In some of these trials, for example, previously frozen fetal tissue-derived NPCs were prepared at a storage facility to a final cell concentration and shipped overnight in hibernation buffer at 4°C to the clinical site. After the viability test of shipped-cell suspension was completed, the cells were directly used for spinal injections^37,39,40^.

### Human iPSCs-Derived NPCs Show Comparable Differentiation After *in Vivo* Grafting as Seen for Fetal Tissue-Derived or Embryonic Cell-Derived NPCs

In our current study, we have demonstrated a comparable grafted-cell survival and differentiation once NPCs were grafted as freshly harvested proliferating NPCs or previously frozen, thawed, and grafted NPCs. Using IF a robust neuronal differentiation was seen with a subpopulation of grafted neurons acquiring inhibitory or excitatory neurotransmitter phenotype at 2–6 months post grafting. The presence of GFAP+ astrocytes and Olig2+oligodendrocytes was also seen in NPCs-grafted striata and spinal cord. Using species-specific mRNA sequencing of human NPCs grafts, we also confirmed the presence of neuronal and glial differentiation-associated transcripts and the lack of endoderm and mesoderm cell derivatives. These data are similar to our previous studies demonstrating the safety and differentiation profile of human ES-derived NPCs or porcine skin fibroblasts-derived iPSC-NPCs grafted spinally as xenografts or allogeneic or syngeneic grafts^24,49^. Comparable long-term survival and differentiation of human NPCs derived from fetal tissue, ES lines, or iPSC line were demonstrated in naive animals^43,50^ or in several animal models of neurodegenerative diseases including spinal traumatic injury^7,44,51,52^, spinal ischemic injury^4–6^, stroke^53–58^, brain traumatic injury^59–61^, or ALS^22,44,62^.

Jointly, our current study demonstrates that the use of an NPC manual-selection from human iPSCs and NPCs monolayer-expansion protocol is a highly effective approach in generating sufficient quantities of NPCs which can be stored-frozen and used directly in subsequent *in vivo* grafting studies. Similarly, our data demonstrate that the iPSCs-derived NPCs are equivalent (as defined by post-grafting differentiation characteristics and lack of tumorigenesis) to the NPC lines derived from fetal tissue from ES cells.

### The Use of a Novel Human Spinal Cell Injection System Is Safe, Simple to Operate and Deliver Injected Cells Into Intended Spinal Targets in Adult Pig

Previous large animal and clinical studies have employed several spinal injection devices to deliver cells into spinal parenchyma. Conceptually, two different injections systems were used: (1) a hand-held injection needle directly attached to the syringe, or (2) an injection needle/syringe attached to an external frame with or without XYZ manipulator^38,63,64^. The XYZ manipulator is used for syringe/needle positioning and parenchymal injection. While effective in delivering cells into specific spinal cord targets these delivery systems lack the ability to synchronize the needle position with spinal cord pulsation (ie, periodic dorsoventral movement of the spinal cord primarily caused by breathing waves). This may lead, in some cases, to spinal cord injury and/or local bleeding at and around the needle injection tract. More recently a free-floating injection needle was developed where the movement of the spinal cord (ie, pulsation) is effectively eliminated (concerning the position of needle tip). This needle system was successfully used in human ALS and spinal trauma trials^37,39,40^. One of the relative limitations of this system is the need for multiple needle-assembly manipulation steps before each injection can be performed. As such, the system is rather complicated to use and requires a detailed and repetitive training of neurosurgeon(s) to master the injection procedure.

We have developed a new human spinal injection system that is composed of a self-anchoring spinal platform, a fine XYZ manipulator firmly attached to the spinal platform, and a magnetic spring-effect-inducing injection needle. The use of this needle, which follows the dorso-ventral movement of the spinal cord (ie pulsation) after the needle is placed into the parenchyma (due to spring effect), effectively eliminates a potential needle-induced spinal cord injury caused by the spinal cord pulsation. The second important feature of the injection system is the simplicity of its operation while performing multiple-repetitive injections. After the injection needle is attached to the XYZ manipulator, a one-hand operation is only required to control the motion of the XYZ manipulator in all three axes. As such, repetitive injection can be performed with high safety and simplicity and can easily be completed by any trained neurosurgeon with the expertise in performing spinal laminectomy and durotomy. In our current safety study, using this injection device in adult pigs, we have performed a total of 10 bilateral injections of NPCs. This injection design (ie the number and location of injections delivered) is similar to the one we have recently used in competed Phase I human spinal trauma trial where 6 injections were delivered around the injury epicenter^37^ or in ALS patients receiving lumbar and/or cervical NPCs grafts^39,40^. In the pigs injected using our new injection device, no cell-injection procedure-related side effect (such as motor weakness or allodynia) was seen if assessed between 24 and 48 h after cell delivery and with grafted cells identified in the targeted gray matter region. Jointly, these data demonstrate that this injection system can effectively and safely be used in future human clinical trials designed to deliver cells into spinal parenchyma (gray or white mater).

In summary, we have demonstrated a successful generation of human iPSCs-derived NPCs cell lines by using an NPC manual-selection protocol and expansion of cells in a monolayer cell culture. *In vivo* grafted previously frozen and washed NPCs show long-term engraftment, neuronal and glial differentiation, and with the lack of tumor formation. The use of a novel human spinal “magnetic” injection needle with a simple operation design was shown to deliver NPCs into adult pig spinal cord with no detectable injection-procedure-related side effect. Accordingly, the use of hiPSCs-NPCs generation protocol and human injection device may represent an additional cell source and clinical cell delivery system to be considered in perspective human spinal cell-replacement clinical trials.

## Supplemental Material

sj-jpg-1-cll-10.1177_09636897221107009 – Supplemental material for Expandable Sendai-Virus-Reprogrammed Human iPSC-Neuronal Precursors: In Vivo Post-Grafting Safety Characterization in Rats and Adult Pig Click here for additional data file. Supplemental material, sj-jpg-1-cll-10.1177_09636897221107009 for Expandable Sendai-Virus-Reprogrammed Human iPSC-Neuronal Precursors: In Vivo Post-Grafting Safety Characterization in Rats and Adult Pig by Yoshiomi Kobayashi, Michiko Shigyo, Oleksandr Platoshyn, Silvia Marsala, Tomohisa Kato, Naoki Takamura, Kenji Yoshida, Akiyoshi Kishino, Mariana Bravo-Hernandez, Stefan Juhas, Jana Juhasova, Hana Studenovska, Vladimir Proks, Shawn P. Driscoll, Thomas D. Glenn, Samuel L. Pfaff, Joseph D. Ciacci and Martin Marsala in Cell Transplantation

sj-jpg-2-cll-10.1177_09636897221107009 – Supplemental material for Expandable Sendai-Virus-Reprogrammed Human iPSC-Neuronal Precursors: In Vivo Post-Grafting Safety Characterization in Rats and Adult Pig Click here for additional data file. Supplemental material, sj-jpg-2-cll-10.1177_09636897221107009 for Expandable Sendai-Virus-Reprogrammed Human iPSC-Neuronal Precursors: In Vivo Post-Grafting Safety Characterization in Rats and Adult Pig by Yoshiomi Kobayashi, Michiko Shigyo, Oleksandr Platoshyn, Silvia Marsala, Tomohisa Kato, Naoki Takamura, Kenji Yoshida, Akiyoshi Kishino, Mariana Bravo-Hernandez, Stefan Juhas, Jana Juhasova, Hana Studenovska, Vladimir Proks, Shawn P. Driscoll, Thomas D. Glenn, Samuel L. Pfaff, Joseph D. Ciacci and Martin Marsala in Cell Transplantation

sj-jpg-3-cll-10.1177_09636897221107009 – Supplemental material for Expandable Sendai-Virus-Reprogrammed Human iPSC-Neuronal Precursors: In Vivo Post-Grafting Safety Characterization in Rats and Adult Pig Click here for additional data file. Supplemental material, sj-jpg-3-cll-10.1177_09636897221107009 for Expandable Sendai-Virus-Reprogrammed Human iPSC-Neuronal Precursors: In Vivo Post-Grafting Safety Characterization in Rats and Adult Pig by Yoshiomi Kobayashi, Michiko Shigyo, Oleksandr Platoshyn, Silvia Marsala, Tomohisa Kato, Naoki Takamura, Kenji Yoshida, Akiyoshi Kishino, Mariana Bravo-Hernandez, Stefan Juhas, Jana Juhasova, Hana Studenovska, Vladimir Proks, Shawn P. Driscoll, Thomas D. Glenn, Samuel L. Pfaff, Joseph D. Ciacci and Martin Marsala in Cell Transplantation
