# Supplementary figures and images for: Expandable Sendai-Virus-Reprogrammed Human iPSC-Neuronal Precursors: In Vivo Post-Grafting Safety Characterization in Rats and Adult Pig
Source: Cell Transplant. 2023 Apr 23;32:09636897221107009. doi: 10.1177/09636897221107009 (PMC10134149; doi:10.1177/09636897221107009)

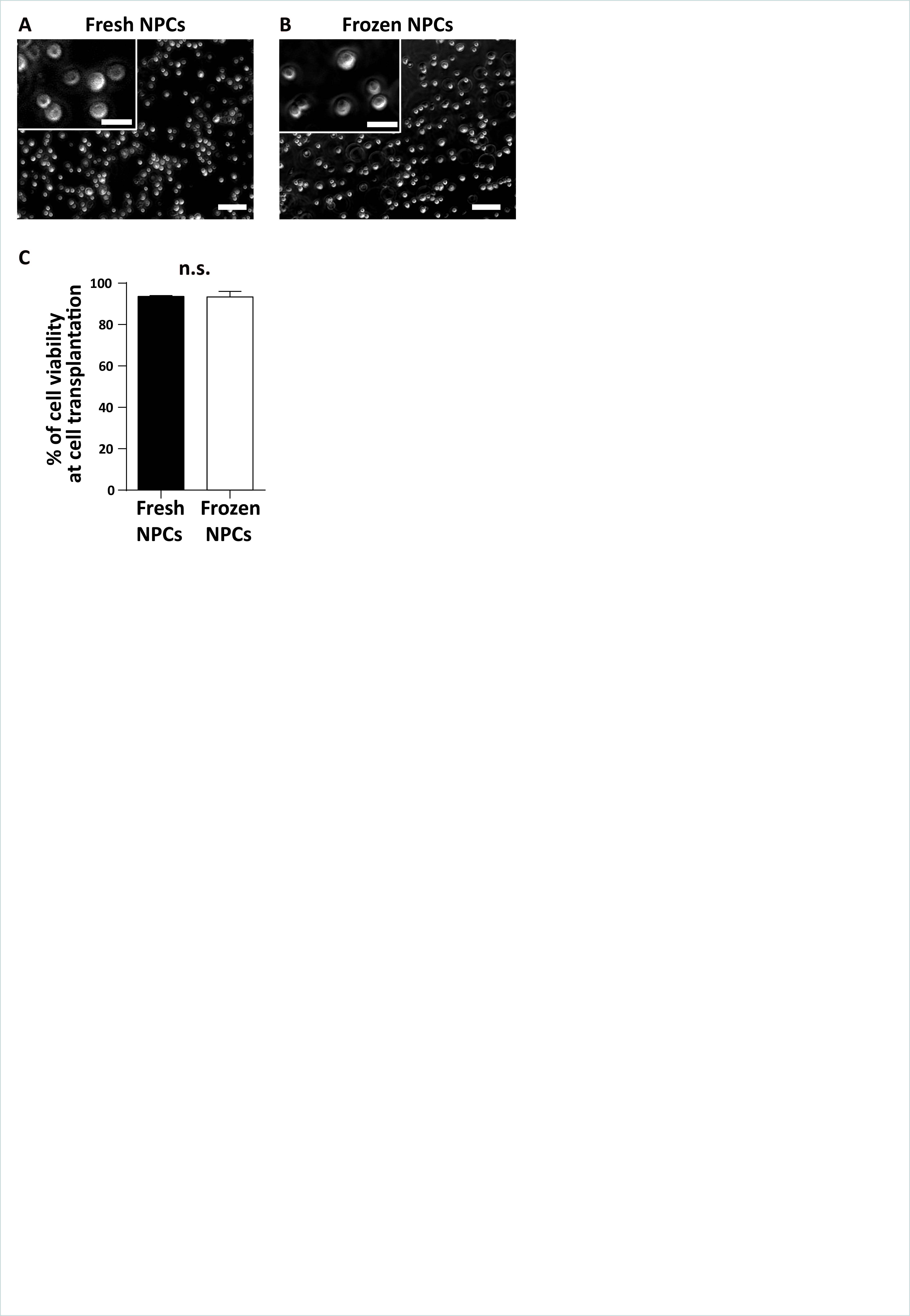

Supplement: sj-jpg-1-cll-10.1177_09636897221107009 – Supplemental material for Expandable Sendai-Virus-Reprogrammed Human iPSC-Neuronal Precursors: In Vivo Post-Grafting Safety Characterization in Rats and Adult Pig [file sj-jpg-1-cll-10.1177_09636897221107009.jpg]

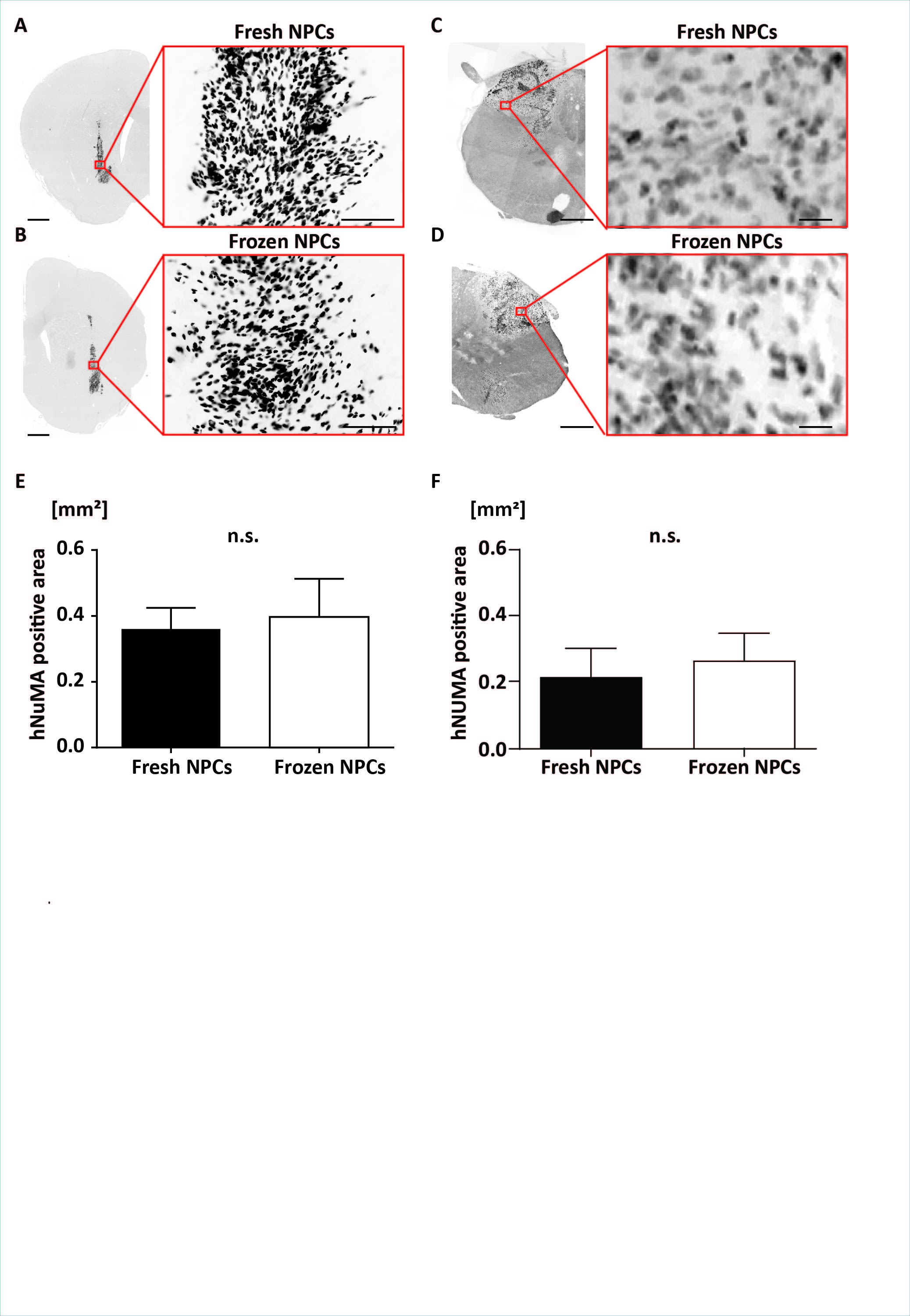

Supplement: sj-jpg-2-cll-10.1177_09636897221107009 – Supplemental material for Expandable Sendai-Virus-Reprogrammed Human iPSC-Neuronal Precursors: In Vivo Post-Grafting Safety Characterization in Rats and Adult Pig [file sj-jpg-2-cll-10.1177_09636897221107009.jpg]

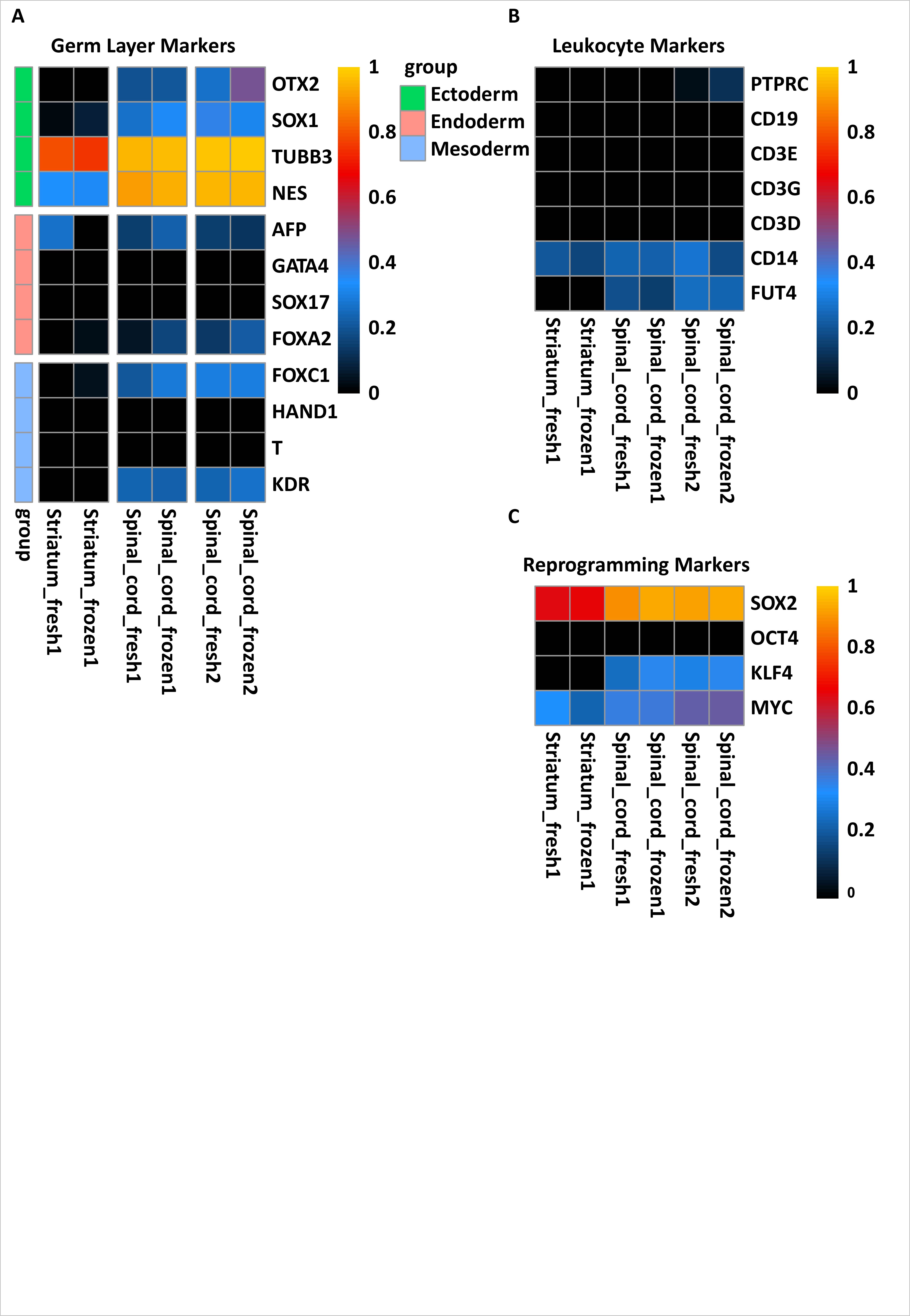

Supplement: sj-jpg-3-cll-10.1177_09636897221107009 – Supplemental material for Expandable Sendai-Virus-Reprogrammed Human iPSC-Neuronal Precursors: In Vivo Post-Grafting Safety Characterization in Rats and Adult Pig [file sj-jpg-3-cll-10.1177_09636897221107009.jpg]
